# Multiple Feature Selection Strategies Identified Novel Cardiac Gene Expression Signature for Heart Failure

**DOI:** 10.3389/fphys.2020.604241

**Published:** 2020-11-11

**Authors:** Dan Li, Hong Lin, Luyifei Li

**Affiliations:** ^1^ Department of Cardiovascular Medicine, First Hospital Affiliated to Harbin Medical University, Harbin, China; ^2^ Internal Medicine-Cardiovascular Department, Harbin Chest Hospital, Harbin, China

**Keywords:** heart failure, microarray, biomarker, network, molecular mechanism

## Abstract

Heart failure (HF) is a serious condition in which the support of blood pumped by the heart is insufficient to meet the demands of body at a normal cardiac filling pressure. Approximately 26 million patients worldwide are suffering from heart failure and about 17–45% of patients with heart failure die within 1-year, and the majority die within 5-years admitted to a hospital. The molecular mechanisms underlying the progression of heart failure have been poorly studied. We compared the gene expression profiles between patients with heart failure (*n* = 177) and without heart failure (*n* = 136) using multiple feature selection strategies and identified 38 HF signature genes. The support vector machine (SVM) classifier based on these 38 genes evaluated with leave-one-out cross validation (LOOCV) achieved great performance with sensitivity of 0.983 and specificity of 0.963. The network analysis suggested that the hub gene *SMOC2* may play important roles in HF. Other genes, such as *FCN3*, *HMGN2*, and *SERPINA3*, also showed great promises. Our results can facilitate the early detection of heart failure and can reveal its molecular mechanisms.

## Introduction

Heart failure (HF) is a serious condition in which the support of blood pumped by the heart is insufficient to meet the demands of body at a normal cardiac filling pressure (Ramachandra et al., 2020). Defined as a syndrome with high morbidity and mortality, HF is the major cause of death and a serious threat to human health for a long period (Jarcho, 2020). Approximately 26 million patients worldwide are suffering from heart failure, and the society faces the long-term great stresses on patients, medical stuff, and medical systems (Bowen et al., 2020). About 17–45% of patients with heart failure die within 1 year, and the majority die within 5 years admitted to a hospital in worldwide (Davison and Cotter, 2015; Zhou et al., 2020). However, the survival rates for patients with HF have improved in many parts of the world in recent years along with the advanced therapies and patient management systems. Heart failure is a complex disease, and so many factors are responsible that it is hard to blame it on one specific issue (McMurray and Pfeffer, 2005).

Over the past decades, the genetic causes and molecular mechanism underlying the progression of heart failure have been partially illustrated. Most previous studies in heart failure are limited by inadequate biological samples from patients with heart failure (Prohászka et al., 2013). Since then, studies have focused on the molecular mechanism of heart failure by virtue of animal models in combination with molecular biological techniques. Previous studies suggested that classification of disease status for HF is much important for the decision of treatment and improvement of prognosis (van Oort et al., 2011). They have discovered that novel gene biomarkers play a vital role in various diseases depending on the leapfrog development of RNA-Seq technology (Asakura and Kitakaze, 2009). According to previous reports, the specific gene expression is related to the pathological conditions of HF.

Liu et al. (2015) collected six samples from three controls, one ischemic heart disease (ISCH), and two dilated cardiomyopathies (DCMs) and used RNA-Seq to filter novel gene signatures for HF, and precisely categorize HF status in larger samples of 313 patients. Vigil-Garcia et al. (2020) selected novel genes induced during pathological cardiac hypertrophy that are relevant for human HF through cardiomyocyte-specific gene expression analysis. These results recognized PFKP as a novel potential therapeutic target to prohibit the succession of HF. Tan et al. (2002) used microarrays to describe gene expression fingerprints of HF etiologies based on seven non-failing human hearts and eight failing human hearts with a diagnosis of end-stage dilated cardiomyopathy. Zhou et al. (2020) proposed that valosin-containing protein could protect the heart against pressure overload-induced heart failure using RNA-Seq and a comprehensive bioinformatics analysis. Kittleson et al. (2004) used microarrays of 48 myocardial samples and gene expression profiling to predict biomarkers in determining prognosis and response to therapy in HF precisely. All these studies were based on microarrays, which have been the remarkable method for gene expression studies because of their ability to filter thousands of transcripts.

In our study, we tried to detect the novel HF signature genes and their networks from previous transcriptomic data which included the gene expression profiles in patients with heart failure (*n* = 177) and without heart failure (*n* = 136) using advanced bioinformatics methods. Compared with previous studies, which are intended to find the biomarker for HF put the focus on separated gene, our study focused on the linkage among them. We built the support vector machine (SVM) model with the application of multiple feature selection methods: Monte Carlo Feature Selection (MCFS; Draminski et al., 2008; Chen et al., 2018a, 2020; Pan et al., 2019b; Li et al., 2020a) and incremental feature selection (IFS; Zhang et al., 2016; Chen et al., 2018b, 2020; Wang et al., 2018; Pan et al., 2019a). What is more, we used the Search Tool for the Retrieval of Interacting Genes (STRING) database (Szklarczyk et al., 2018) to explore the protein interaction networks. A remarkable result of our study is that 38 selected genes can serve as novel biomarkers for HF and can conduce to revealing the pathological mechanism of HF.

## Materials and Methods

### The Microarray Data of Heart Failure Patients

We downloaded the microarray gene expression data of 177 patients with heart failure and 136 patients without heart failure from Gene Expression Omnibus (GEO) at https://www.ncbi.nlm.nih.gov/geo/query/acc.cgi?acc=GSE57338 (Liu et al., 2015). The expression levels of 33,297 probes corresponding to 20,254 genes in the cardiac tissue were measured with Affymetrix Human Gene 1.1 ST Array. The probes corresponding to the same gene were averaged to obtain the gene expression levels, and the gene expression levels were quantile normalized using function normalize.quantiles from R/Bioconductor package preprocessCore^1^ to minimize the systematic variance. The normalized data were used for further feature selections.

### Select the Genes Based on Their Importance to Classify the Heart Failure Patients

There have been many methods for identifying differentially expressed genes (DEGs), such as *t*-test. But such methods only consider the distribution of one gene each time, and do not consider the relationship among genes (Tao et al., 2020). That leads to two limitations: (1) The distribution difference of a gene is not equivalent to its classification ability; and (2) The combinations of the most significant DEGs may not have good performance since they may be redundant and do not help each other to achieve a better performance. Therefore, we adopted machine learning based multiple feature selection strategies to objectively select the optimal heart failure signature. The machine learning-based methods have been widely used and achieved great success in biomarker discovery (Wang and Huang, 2019; Li et al., 2020a,b; Yuan et al., 2020; Zhang et al., 2020a,b; Zhu et al., 2020).

**Figure 1:**
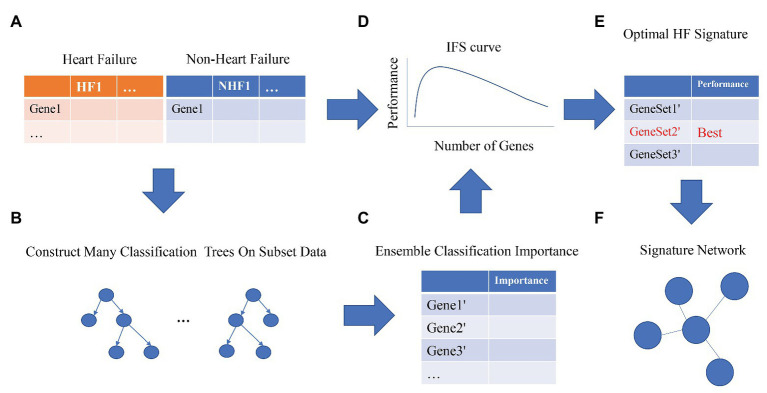
The workflow for optimal heart failure signature identification. The workflow integrated the strategies of Monte Carlo Feature Selection (MCFS) and incremental feature selection (IFS). Step **(A)**: data preprocessing. Steps **(B,C)**: MCFS. Steps **(D,E)**: IFS. Step **(F)**: signature network.

The proposed multiple feature selection strategies can be summarized as Figure 1. First, the expression profiles of 20,254 genes in 177 patients with heart failure and 136 patients without heart failure were normalized. Second, we randomly selected many subset data to construct the classification trees using Monte Carlo strategy (Draminski et al., 2008; Chen et al., 2018a, 2020; Pan et al., 2019b; Li et al., 2020a). To perform MCFS, we used the dmLab software version 2.3.0 from https://home.ipipan.waw.pl/m.draminski/mcfs.html. Third, all these trees were ensembled to calculate the classification importance of the genes. The important genes should appear in a large number of trees and be able to correctly classify the samples into right groups. Fourth, the top ranked genes (1,000 in this study) were further analyzed using IFS strategy (Zhang et al., 2016; Chen et al., 2018b, 2019; Wang et al., 2018; Pan et al., 2019a). Each time, a gene set including the top *K* most important genes (*K* = 1, 2, 3, …, 1,000) was used to train a SVM model, and its performance was evaluated with leave-one-out cross validation (LOOCV; Li and Huang, 2018). To build the SVM, we used the function svm from R package e1071.^2^ Fifth, the optimal heart failure signature was the gene set with the best performance. If the IFS curve did not reach its peak or the plateau area and kept increasing as the number of genes increased, more top genes should be analyzed. Sixth, to better understand the underlying regulatory mechanisms of the signature and increase the interpretability of the signature, we constructed the signature network based on STRING database version 11.0 (http://string-db.org; Szklarczyk et al., 2018; Shi et al., 2020).

## Results

### The Optimal Heart Failure Signature Identification

**Figure 2:**
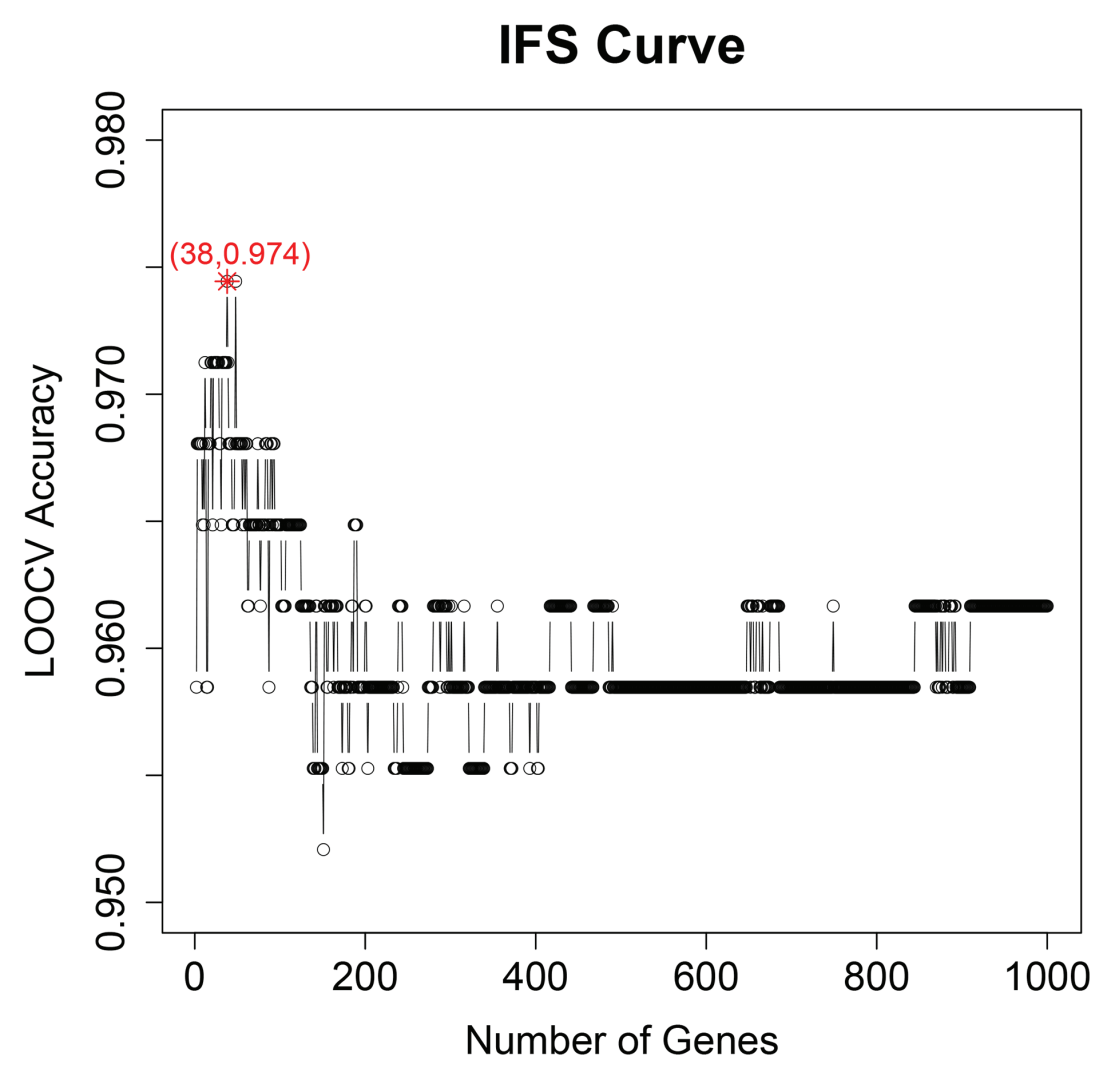
The IFS curve of optimal heart failure signature identification. It showed the relationship between the number of genes (*x*) and their LOOCV accuracy (*y*). The peak accuracy was 0.974 when 38 genes were used. Therefore, the 38 genes were chosen as optimal heart failure signature.

We adopted multiple feature selection strategies (Figure 1) to identify the optimal heart failure signature. It integrated the strategies of MCFS and IFS. Step A was data preprocessing. MCFS included Steps B and C. IFS included Steps D and E. Step F was to interpret the biological mechanisms of the signature. As demonstrated in Figure 1D, the actual IFS curve was shown in Figure 2. The highest LOOCV accuracy was 0.974 when the top 38 MCFS genes were used to train the SVM model. Therefore, these 38 genes were chosen as the optimal heart failure signature, which was shown in Table 1. The confusion matrix of the 38 optimal heart failure signature genes which compared the actual class labels and precited class labels of all samples were given in Table 2. Their LOOCV sensitivity, specificity, and accuracy were 0.983, 0.963, and 0.974, respectively. The performance was great.

**Table 1:** The 38 optimal heart failure (HF) signature genes.

Rank	Gene symbol	Full name	Importance
1	HMGN2	High mobility group nucleosomal binding domain 2	0.571
2	HMOX2	Heme oxygenase 2	0.527
3	SERPINA3	Serpin family A member 3	0.499
4	TUBA3D	Tubulin alpha 3d	0.489
5	ECM2	Extracellular matrix protein 2	0.481
6	FREM1	FRAS1 related extracellular matrix 1	0.461
7	FCN3	Ficolin 3	0.458
8	ZMAT1	Zinc finger matrin-type 1	0.405
9	SMOC2	SPARC related modular calcium binding 2	0.386
10	CSDC2	Cold shock domain containing C2	0.383
11	LCN6	Lipocalin 6	0.359
12	LUM	Lumican	0.356
13	FURIN	Furin, paired basic amino acid cleaving enzyme	0.349
14	LAD1	Ladinin 1	0.338
15	MNS1	Meiosis specific nuclear structural 1	0.338
16	ASPN	Asporin	0.317
17	FRZB	Frizzled related protein	0.310
18	GGT5	Gamma-glutamyltransferase 5	0.296
19	TUBA3E	Tubulin alpha 3e	0.293
20	PDE5A	Phosphodiesterase 5A	0.292
21	ISLR	Immunoglobulin superfamily containing leucine rich repeat	0.289
22	S1PR3	Sphingosine-1-phosphate receptor 3	0.279
23	SFRP4	Secreted frizzled related protein 4	0.271
24	APBB3	Amyloid beta precursor protein binding family B member 3	0.270
25	USP31	Ubiquitin specific peptidase 31	0.268
26	SLCO4A1	Solute carrier organic anion transporter family member 4A1	0.251
27	VSIG4	V-set and immunoglobulin domain containing 4	0.251
28	KCNN3	Potassium calcium-activated channel Subfamily N member 3	0.250
29	FAM58A	CCNQ cyclin Q cyclin Q	0.248
30	AP3M2	Adaptor related protein complex 3 subunit mu 2	0.247
31	C15orf59	INSYN1 inhibitory synaptic factor 1	0.243
32	BTN3A1	Butyrophilin subfamily 3 member A1	0.243
33	ZDHHC16	Zinc finger DHHC-type containing 16	0.241
34	CD163	CD163 molecule	0.238
35	SEMA4B	Semaphorin 4B	0.237
36	ST6GALNAC3	ST6 N-acetylgalactosaminide alpha-2,6-sialyltransferase 3	0.228
37	TTC3	Tetratricopeptide repeat domain 3	0.228
38	MATN2	Matrilin 2	0.219

**Table 2:** The confusion matrix of the 38 optimal heart failure signature genes.

	Predicted HF	Predicted NHF
Actual HF	174	3
Actual NHF	5	131

### The Expression Pattern of the 38 Genes in Patients With HF and Without HF

**Figure 3:**
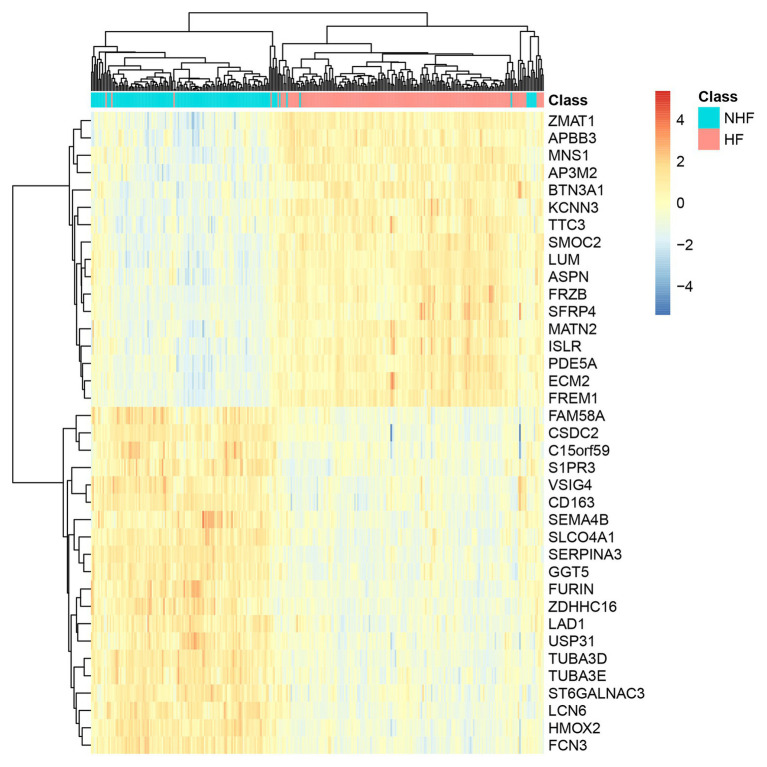
The heatmap of the 38 genes in 177 HF and 136 non-heart failure (NHF) patients. Most samples were clustered into the correct groups. Only very few samples were misclustered. Within the 38 genes, 17 genes were highly expressed in HF, and 20 genes were lowly expressed in HF.

We plotted the heatmap of the 38 genes in 177 patients with heart failure (HF) and 136 patients without heart failure [non-heart failure (NHF)] in Figure 3. It can be seen that most samples were clustered into the correct groups. Only very few samples were misclustered. Within the 38 genes, 17 genes (*ZMAT1*, *APBB3*, *MNS1*, *AP3M2*, *BTN3A1*, *KCNN3*, *TTC3*, *SMOC2*, *LUM*, *ASPN*, *FRZB*, *SFRP4*, *MATN2*, *ISLR*, *PDE5A*, *ECM2*, and *FREM1*) were highly expressed in HF and 20 genes (*FAM58A*, *CSDC2*, *C15orf59*, *S1PR3*, *VSIG4*, *CD163*, *SEMA4B*, *SLCO4A1*, *SERPINA3*, *GGT5*, *FURIN*, *ZDHHC16*, *LAD1*, *USP31*, *TUBA3D*, *TUBA3E*, *ST6GALNAC3*, *LCN6*, *HMOX2*, and *FCN3*) were lowly expressed in HF.

### The Network of the 38 Genes

**Figure 4:**
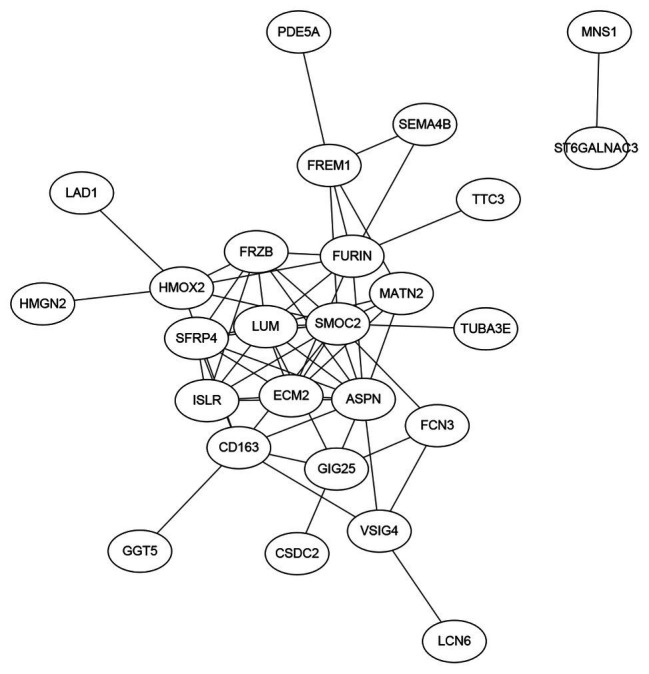
The network of the 38 genes searched on the Search Tool for the Retrieval of Interacting Genes (STRING) database. We searched the interactions of the 38 genes on the STRING database and plotted the network. It can be seen that *SMOC2* located in the hub position of the network and its functions in HF was intensively studied.

Signature genes were not necessarily key regulators. They could be only markers. But if the signature genes have clear biological functions, they certainly can be better interpreted. Therefore, as we stated in Figure 1F, we searched the interaction among the STRING database (https://string-db.org/; Szklarczyk et al., 2018) and plotted the networks of the 38 genes in Figure 4. It can be seen that *SMOC2* is located in the hub position of the network.

*SMOC2*, a member of the SPARC family, which is highly expressed during embryogenesis and wound healing. Previous studies recognized that inflammatory pathways were generally dysregulated in right ventricular failure (RVF) tissue. Williams et al. (2018) analyzed mRNA datasets of human non-failing and failing heart samples from patients, and concluded that *SMOC2* was differentially expressed. *SMOC2* could be a potential significance factor that altered remodeling and inflammation for further study in the mechanism of HF. Laugier et al. (2017) found that *SMOC2*, involved in matrix remodeling, is potentially associated with the increased T-helper 1 cytokine-mediated inflammatory damage in heart, using genome-wide cardiac DNA methylation on global gene expression in myocardial samples in chronic Chagas disease cardiomyopathy, which is an inflammatory cardiomyopathy presenting with heart failure and arrhythmia.

## Discussion

In the present study, 38 genes were selected from our prediction model of SVM, implying strong relevance with the pathological mechanisms of HF. After literature retrieval and utilization, several evidences and analysis results have been retrieved to validate the dependability and reality of our analysis.

*FCN3*, a member of ficolin/opsonin p35 lectin family which consists of a collagen-like domain and a fibrinogen-like domain, which were found in all human serum. Prohászka et al. (2013) reported that the main initiator molecules of the lectin complement pathway *MBL*, *FCN2*, and *FCN3* were related to chronic heart failure (CHF). Low *FCN3* levels were related to decreased concentrations of complement factor C3 and increased complement activation product C3a (Prohászka et al., 2013). They also provided evidence for a significant association of low *FCN3* levels with advanced HF and outcome (Prohászka et al., 2013). *FCN3* is reported to be increased in microvesicles obtained from activated platelets and abdominal aortic aneurysm (AAA) tissue (Fernandez-García et al., 2017). There is an obvious relationship between increased *FCN3* plasma levels and AAA presence and progression.

*HMGN2* binds nucleosomal DNA and is associated with transcriptionally active chromatin, which is the top-ranked feature recognized by our bioinformatics analysis. HMGN protein family could regulate chromatin structure and could influence epigenetic modifications. *HMGN2* regulates active and bivalent genes by promoting an epigenetic landscape of active histone modifications at promoters and enhancers (Garza-Manero et al., 2019). *HMGN2* protected corticogenesis *via* maintaining global chromatin accessibility at promoter regions, thus ensuring proper transcriptome regulation (Apelt et al., 2020; Gao et al., 2020). There are few studies to certificate the role of HMGN2 in the progress of HF.

*SERPINA3* also called Alpha-1-Antichymotrypsin or ACT, is first discovered as a plasma protease inhibitor and a member of the serine protease inhibitor (Jiang et al., 2020). Previous study showed that *SERPINA3* emerged as a responsible cardiac secreted factor that is increased in HF patients could be the most robust and promising culprit and were related to long-term mortality. Additionally, several researches thought that mineralocorticoid receptor antagonists (MRAs) were associated to *SERPINA3* (Meijers et al., 2018). Gene expression of *SERPINA3* was significantly increased in the HF group. In circulating plasma, the level of *SERPINA3* in the HF group was confirmed significant increase by ELISA analysis. These results suggested that *SERPINA3* might play an important role in the progression of HF (Zhao et al., 2020). Asakura and Kitakaze (2009) proved that *SERPINA3* might become novel diagnostic and therapeutic targets linked to the pathophysiology of HF using seven microarray datasets previously reported.

Due to the length limitation of the article, we cannot describe all 38 selected genes in detail. After detailed literature review, we found that all the above-mentioned genes play a vital role in the progression of HF, which also verifies the reliability of our prediction model. We believe that these 38 selected genes are meaningful in the development of HF. They will contribute to the study of molecular mechanism, diagnosis, and treatment of HF, and will play an enlightening role in the future molecular biology research.

## Data Availability Statement

The original contributions presented in the study are included in the article/supplementary material, further inquiries can be directed to the corresponding author.

## Author Contributions

DL: conception and design, administrative support, and provision of study materials or patients. HL: collection and assembly of data. HL and LL: data analysis and interpretation. All authors contributed to the article and approved the submitted version.

### Conflict of Interest

The authors declare that the research was conducted in the absence of any commercial or financial relationships that could be construed as a potential conflict of interest.

## Footnotes

^1^https://bioconductor.org/packages/preprocessCore/

^2^https://CRAN.R-project.org/package=e1071
